# Thermomechanical Process Simulation and Experimental Verification for Laser Additive Manufacturing of Inconel^®^718

**DOI:** 10.3390/ma16072595

**Published:** 2023-03-24

**Authors:** Muhammad Qasim Zafar, Jinnan Wang, Zhenlin Zhang, Chaochao Wu, Haiyan Zhao, Ghulam Hussain, Ninshu Ma

**Affiliations:** 1 State Key Laboratory of Tribology, Department of Mechanical Engineering, Tsinghua University, Beijing 100084, China; 2 Faculty of Mechanical Engineering, Ghulam Ishaq Khan Institute of Engineering Sciences and Technology, Topi 23640, Pakistan; 3 Mechanical Engineering Department, College of Engineering, University of Bahrain, Isa Town 32038, Bahrain; 4 Joining and Welding Research Institute, Osaka University, Osaka 567-0047, Japan

**Keywords:** additive manufacturing, laser cladding, FEM-hybrid simulation, Inconel^®^718, residual stress, geometric distortion, X-ray diffraction

## Abstract

Laser cladding has emerged as a promising technique for custom-built fabrications, remanufacturing, and repair of metallic components. However, frequent melting and solidification in the process cause inevitable residual stresses that often lead to geometric discrepancies and deterioration of the end product. The accurate physical interpretation of the powder consolidation process remains challenging. Thermomechanical process simulation has the potential to comprehend the layer-by-layer additive process and subsequent part-scale implications. Nevertheless, computational accuracy and efficacy have been serious concerns so far; therefore, a hybrid FEM scheme is adopted for efficient prediction of the temperature field, residual stress, and distortion in multilayer powder-fed laser cladding of Inconel^®^718. A transient material deposition with powder material modeling is schematized to replicate the fabrication process. Moreover, simulation results for residual stress and distortion are verified with in-house experiments, where residual stress is measured with XRD (X-Ray Diffraction) and geometric distortion is evaluated with CMM (Coordinate Measuring Machine). A maximum tensile residual stress of 373 ± 5 MPa is found in the vicinity of the layer right in the middle of the substrate and predicted results are precisely validated with experiments. Similarly, a 0.68 ± 0.01 mm distortion is observed with numerical simulation and showed a precise agreement with experimental data for the same geometry and processing conditions. Conclusively, the implemented hybrid FEM approach demonstrated a robust and accurate prediction of transient temperature field, residual stresses, and geometric distortion in the multilayer laser cladding of Inconel^®^718.

## 1. Introduction

**Figure 1:**
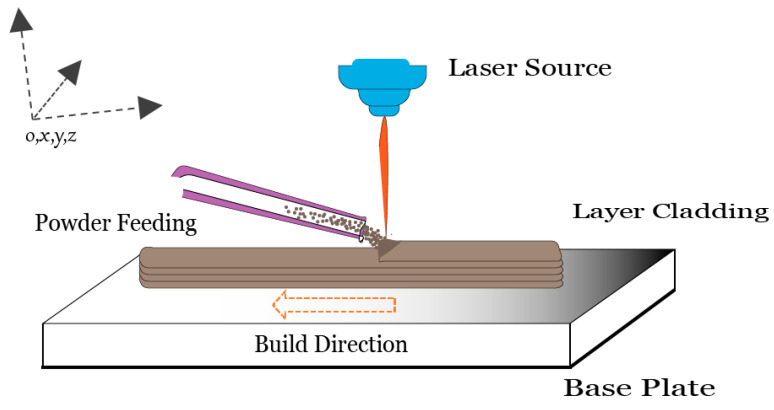
Powder-fed laser cladding process schematic.

Additive manufacturing (AM) is a relatively new process to fabricate three-dimensional, complex, and customized components in successive layers and has been established as a viable fabrication technique in the current manufacturing realm [1]. This state-of-the-art technology is even envisioned to add “Time” as its fourth dimension, formally known as “4D Printing” [2]. Metal additive manufacturing is forecasted as a future of customized fabrication, especially for biomedical, automotive, and aerospace components [1]. Directed energy deposition (DED), particularly laser cladding, has been practiced widely due to its rapid fabrication, feedstock flexibility, higher deposition rate, and comparable mechanical properties. A small heat-affected zone (HAZ) with a precise deposition scheme makes the process more suitable than arc welding, plasma spraying, and flame spraying [3]. Laser cladding is an appropriate technique for engraving additional features on existing components and it has been undertaken in the repair, maintenance, and remanufacturing of in-service components [4]. Laser cladding can process multi-materials simultaneously with adequate deposition on the control feed rate for the development of corrosion-free, wear-resistant heterogeneous structures [5]. The typical process involves a concentrated laser source impinging on a coaxial powder stream to consolidate a thin layer on a previously deposited material, as illustrated in Figure 1. The associated localized heating, subsequent melting, and solidification of powder stimulate thermal residual stresses, which often lead to geometric inaccuracies and premature fracture [1,6]. Understanding of thermomechanical behavior is very critical for the processing of Inconel^®^718 as it provokes residual stresses, distortion, coarse microstructure, microsegregation of elements, and encourages unstable phases. Thermomechanical phenomena become more complex when multilayer consolidation is involved, especially in powder-based additive manufacturing. Eventually, thermomechanical repercussions yields residual stresses and if the residual stresses are too high, the material may lead to compromised dimensional accuracy, part-scale distortion, cracking, or even catastrophic failure [7]. Several factors influence the development of residual stresses during processing, e.g., process parameters, material properties including thermal expansion and contraction, and geometrical design [8]. Noteworthily, laser cladding is sensitive to process parameters, which should be handled carefully for precise and accurate fabrications.

The traditional hit-and-trial approach is expensive and time-consuming; however, numerical simulation has expedited the process and performance comprehension for additively manufactured components [9,10,11]. Persuasive thermomechanical simulation depends on the accuracy of the numerical model, mesh convergency, material modeling, heat source configuration, and boundary conditions [7,12]. Previous studies have shown that the finite element method (FEM) is unanimously accepted as a reliable technique to interpret additive process physics on a part scale; however, high computational cost is a primary hindrance in the widespread adaptation of FEM simulation [9,13]. Laser cladding of Nickel-based superalloys is moving towards several industrial applications, particularly in the manufacturing of gas turbine, aviation, and aerospace components because of their good corrosion resistance, fracture toughness, oxidation resistance, as well as high-service-temperature applications [14,15,16,17]. Controllable energy input and moderate heat intensity make the laser cladding process reasonable for the repair and maintenance of surface-based defects in engineering components fabricated with Nickel-based superalloys [18]. Among all, Inconel^®^718 has become one of the widely used materials owing to its superior fatigue and creep properties, along with exceptional corrosion resistance at elevated temperatures up to 650 °C [19]. The Inconel^®^718 is strengthened by a body-centered tetragonal phase of γ″ (Ni3Nb) and an intermetallic phase of γ’ (Ni3(Al, Ti)). Nevertheless, inadequate processing may lead to carbides and laves phase formation, which is detrimental to the strength and stability of Inconel^®^718 [20].

Serious efforts are underway to boost numerical efficacy by developing novel numerical algorithms and schemes [9]. An elastic–plastic model was developed in ABAQUS^®^ to investigate the thermomechanical behavior of the laser cladding process with quasi-static modeling to save computational costs [21]. Murakawa et al. [22] proposed an inherent strain-based iterative substructure method (ISM) method for the calculation of welding distortion that is 5–10 times faster than commercial code. Further, it was combined with dynamic mesh refinement for residual stress and distortion of the wire arc additive manufacturing process by Huang et al. [23]. That numerical approach demonstrated excellent computational performance compared to ABAQUS^®^. Likewise, an innovative scheme was proposed to compute an effective computation zone as a boundary condition to save calculation time up to 10^4^ of the traditional FEM scheme [24]. Ma et al. [25] presented a computationally efficient parallel computing program “JWRIAN-hybrid” based on a combination of an accelerated explicit and implicit FEM scheme for temperature, residual stress, and distortion prediction in welding structures [25]. The proposed hybrid algorithm has been implemented for welding residual stress estimation previously [26]. Now, it is modified and extended to the simulation with a transient material deposition scheme to replicate physical material consolidation in a typical laser cladding process, as it is believed that material deposition might affect temporal and spatial temperature distribution and subsequent residual stresses and distortion in the multilayer cladding process.

The proposed study presents a thermomechanical process simulation with experimental validation for multilayer laser cladding additive manufacturing of Inconel^®^718 using a novel hybrid FEM simulation algorithm. Ten layers were consolidated on optimum process parameters using an in-house laser cladding facility. The residual stress is measured with XRD and part deflection is quantified with CMM. Likewise, a 3D sequentially coupled FEM simulation is performed with the same geometry, domain size, and experimental conditions in a JWRIAN-hybrid FEM solver. The implemented FEM hybrid approach is significantly efficient because of its explicit and implicit algorithms for the calculation of the thermomechanical process. Powder material modeling and transient material deposition for powder streaming is exclusively considered for the exact replication of the physical process. Finally, FEM simulation results are compared with experimental data to prove the efficacy of the proposed numerical scheme for multilayer cladding of Inconel^®^718.

## 2. Materials and Methods

### 2.1. Material Specifications

**Figure 2:**
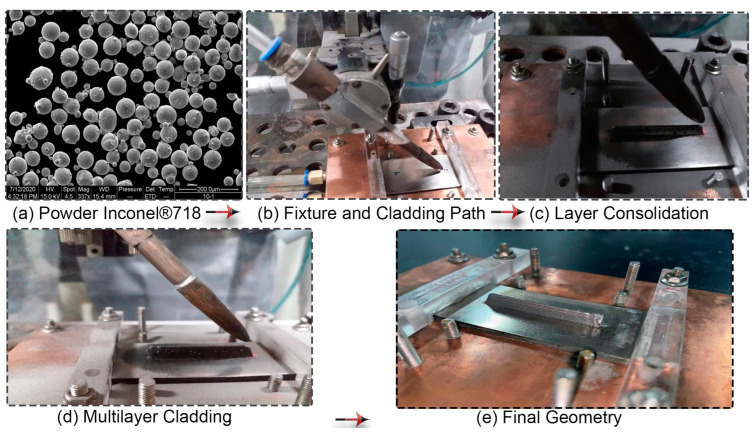
Experimental process sequence from powder to final geometry of 10 layers.

Inconel^®^718 powder particles are produced by the plasma rotating electrode process (PREP). The chemical composition of the powder material used for consolidation is provided in Table 1. Spherical powder particles are dried in a heat treatment oven for 2 h at a constant temperature of 150 °C and the approximate diameter of the powder is in the range of 40 to 85 µm. A microscopic view shows the morphology and quality of Inconel^®^718 particles used in the physical laser cladding process. Additive manufacturing quality may be governed by feedstock material as the flowability and packing density of powder can be affected by the powder size distribution and shape morphology. Small particles may result in agglomeration arising from interparticle forces such as Van der Waals forces. Figure 2a represents a good powder morphology that shows the adequacy of the processing conditions from an input material viewpoint. Stress-free Inconel^®^718 solid plate with a dimension of 70 mm × 30 mm × 3 mm is used as a substrate for multilayer cladding geometry. The substrate material is cleaned with acetone and placed in a heat treatment oven at a constant temperature of 1185 °C for 2 h before laser cladding. Then, the furnace cooled baseplate is clamped from both ends on a purpose-built fixture during laser cladding experiments.

### 2.2. Equipment and Process Detail

Cladding experiments are performed on a paraxial powder-feeding laser cladding platform built in-house, including an IPG YLS-2000 fiber laser device, a DPSF-2 powder-feeding device, and a self-built off-axis laser cladding header. The laser wavelength is 1065 nm and has a circular spot with Gaussian energy distribution. The spot size at the focal point is 2 mm with a 300 mm focal length. The concentrated laser and powder stream intersects to form a continuous melt track on a predefined path with a velocity of 4 mm/s to build the part. The substrate surface is marked accordingly to deposit a 40 mm long clad path with starting and ending positions (see Figure 2b). Optimum laser cladding parameters derived from an experimental campaign and subsequent energy density, scan velocity, and powder streaming combinations, are opted for single-track consolidations, and the corresponding melt pool size is monitored. Melt pool width is approximated with cladding width and depth up to the solidified region in the experimental trial [10]. Fabricated chunks are chosen through several inspection steps. First, fragments are removed from the substrate and properly consolidated chunks are sorted out through visual inspection. Then, selected chunks are further subjected to a detailed metallographic trial with optical microscope (Olympus DP72, Tokyo, Japan) with resolution of 4140 × 3096 pixels, and distinct processing parameters are identified based on high-density solidified tracks. Investigated processing conditions are employed in 10-layer deposition, listed in Table 2.

The protective box is filled with Argon to ensure powder delivery and an inert environment during the cladding process. Ten subsequent layers are consolidated for up to 7 mm of layer height on the baseplate as a complete consolidation process sequence, demonstrated in Figure 2b–e. Finally, the substrate is disassembled from the purpose-built fixture after cooling down to room temperature. The final geometry demonstrates excellent formability and proper consolidation for all 10 layers of Inconel^®^718 deposition, as shown in Figure 2e. Fabrication size width, height, and length are 40 mm, 7 mm, and 2 mm, respectively, measured with a digital vernier caliper (Mitutoyo, Kanagwa, Japan) of 0.01 mm resolution. It is consistent with the CAD dimensions, with a negligible dimensional error on extreme ends.

### 2.3. Experimental Determination of Residual Stress and Distortion

**Figure 3:**
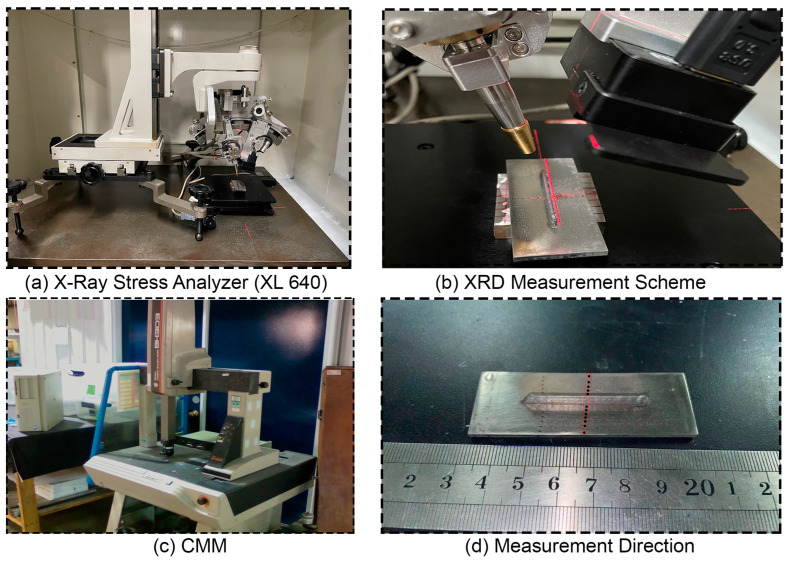
Experimental illustration for residual stress and distortion measurement.

XRD (X-Ray Diffraction) is a non-destructive residual stress measurement technique and a relatively cost-effective and accurate way to quantify surface-level residual stresses in crystalline materials [27]. The magnitude of residual stress can be up to 75% of the nominal yield strength, as reported earlier for additively manufactured components [28]. XRD based on the Sin2ψ method is employed to inspect residual stress in the substrate as per ASTM standard E2860 [29], where interplanar strain in the crystal lattice is quantified and automatically converted to subsequent stress with the latest stress analyzer (XL 640 ST Stress Technologies, Handan, China) equipped with CuKα radiation source. Equipment detail is illustrated in Figure 3a.

The measurements are obtained in the middle of the cross-section right on the substrate surface in the transverse direction, as illustrated in Figure 3. A maximum tensile residual stress of 373 ± 5 MPa is found in the cladding vicinity and a gradually decreasing trend is observed then eventually turned to compressive toward the transverse end of the substrate. Quantitative estimation of substrate distortion is performed with a CMM (BH-303, Mitutoyo, Japan) of 0.0005 mm resolution. The middle section is considered as a reference point and the coordinates of points on the deflected edges of the substrate are mapped; then, measured values are plotted using Origin 8.2. Experimentally, it is observed that the extreme ends are deflected up to 0.68 ± 0.01 mm after unclamping the baseplate from the purpose-built fixture. Moreover, numerical simulation is performed for substrate misalignment and wrappage, and estimated results are validated with in-house experiments.

## 3. Hybrid Numerical Simulation

**Figure 4:**
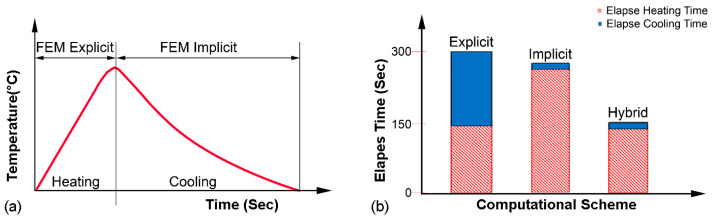
Hybrid FEM simulation. (**a**) Computational scheme in heating and cooling cycle. (**b**) Subsequent elapse time.

Thermo-elastic plastic with implicit FEM scheme has become a generalized approach for the investigation of residual stress and geometric inaccuracies in welding as well as in additive manufacturing; however, large-scale deformation analysis is much more time-consuming. The hybrid FEM scheme opted for a thermomechanical process simulation for 10 layers of the powder-fed laser-assisted cladding process. The hybrid scheme consists of a stable explicit FEM for the calculation of transient thermomechanical phenomena in the heating stage and an implicit FEM for the computation of the cooling step, as nonlinearity becomes weak in the absence of a heat source [26]. The main difference between explicit and implicit analysis is the way in which they handle time-dependent behavior. The explicit analysis is suitable for predicting the dynamic response of a structure under external loads over short time intervals, while the implicit analysis is suitable for predicting the behavior of a structure under steady loads or quasi-static loads over the long term. The hybrid FEM approach is significantly different from the traditional FEM scheme; therefore, subsequent mathematical impressions are included to explain the hybrid formulation for a better understanding of the readers and researchers. Explicit and implicit schemes are presented for the subsequent heating and cooling cycle in Figure 4.

The traditional implicit algorithm is presented here as

(1) K.ΔU=ΔF

It is based on an elastic–plastic theory of material where K, ΔU, and ΔF are the vector of equivalent nodal force increment, nodal displacement increment, and stiffness matrices of the analysis model. When a computer solves this implicit equation, huge power and memory are required to calculate the stiffness matrices K of a large domain in FEM. The explicit algorithm consumes low memory compared to a traditional implicit scheme. A transformed form of the generalized equation of motion as per explicit solution is illustrated here:

(2) $$Ma+C\nu +Ku=F_{ext}$$

(3) $$\left[M\right]\left\{a\left(t+dt\right)\right\}=\left\{F_{ext}\left(t+dt\right)\right\}-\left\{F_{int}\left(t\right)\right\}-\left\{F_{damp}\left(t\right)\right\}$$

where [M], C, K, and $F_{ext}$ are matrices of mass, damping, and stiffness of the nodes, and $\left\{F_{ext}\right\}$, $\left\{F_{int}\right\}$, and $\left\{F_{damp}\right\}$ are the equivalent external nodal, internal, and damping forces, respectively. Time increment dt is small enough to satisfy the Courant–Friedrich–Lewy condition to ensure that information cannot travel more than a certain distance during each time step. This distance is called the “stability distance” and is determined by the wave speed of the system being modeled and the spatial resolution of the numerical method. Courant–Friedrich–Lewy condition is an important criterion that must be satisfied to ensure the accuracy and stability of numerical solutions to partial differential equations:

(4) $$c=\sqrt{\frac{E\left(1-v\right)}{\left(1+v\right)\left(1-2v\right)}\frac{1}{\rho }}$$

where c, ρ, ν, and E are the material constant, density, Poisson’s ratio, and Young’s modulus, respectively, for Inconel^®^718. The accelerated explicit method uses the Accelerated time domain $t^{accelerated}$ to replace the real-time domain $t^{real}$ in the heating and implicit scheme in the cooling process for each layer. JWRIAN-hybrid is based on a combination of explicit and implicit computational algorithms [30]. It has proved to be a more computationally efficient scheme than the commercial FEM solver ABAQUS^®^ [22]. Recently, it has been exercised by Ma et al. [31] for the computation of residual stress and distortion of large welded structures.

### 3.1. FE Model and Mesh

**Figure 5:**
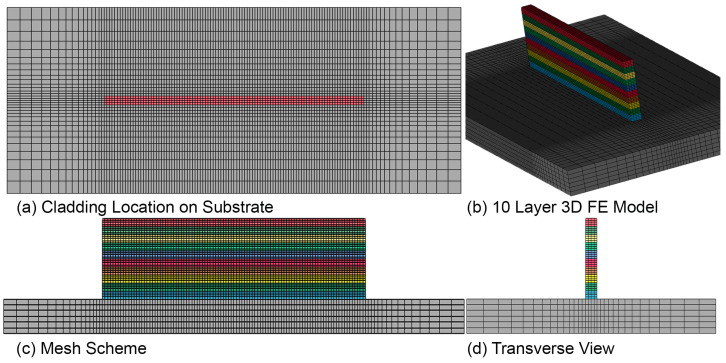
Three-dimensional FEM model with uniform mesh on layers and nonuniform mesh on the substrate.

The development of a 3D FEM model is an essential step in accurately simulating complex structures. In this study, a hexahedral element-based FE model was created with a total of 40,960 elements and 50,461 nodes, as shown in Figure 5. A coarser and irregular mesh scheme was employed for the substrate to reduce the number of elements without compromising accuracy. However, a uniform and fine mesh size of 0.5 mm × 0.5 mm × 0.25 mm was generated for layers and areas of interest on the substrate after conducting a mesh sensitivity analysis. Convergence analysis for size and element type is performed prior to the final simulation in the JWRIAN-hybrid FEM solver.

### 3.2. Material Modeling

Since the material properties change with the temperature variation, solid temperature-dependent thermal and mechanical properties are derived from JMatPro^®^ by considering the materials composition for Inconel^®^718 and are provided in Table 3.

Then, explicit powder properties—e.g., density, thermal conductivity, and emissivity for powder Inconel^®^718—are calculated with a material modeling framework by Zafar et al. [7]. The density of the powder is calculated from its bulk using an underlying expression [32]. Porosity of 32% is assumed to calculate the relative density of streamed powder based on powder packing style, as explained in a previous publication [7].

(5) $$\varphi =\frac{\rho_{solid}-\rho_{powder}}{\rho_{solid}}$$

**Figure 6:**
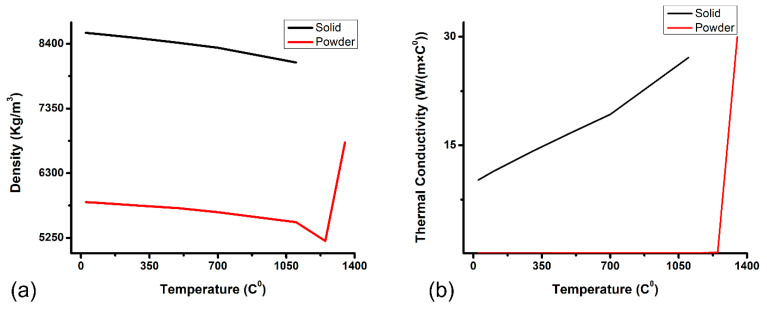
Temperature-dependent material modeling for powder Inconel^®^718. (**a**) Density. (**b**) Thermal conductivity.

Thermal conductivity is calculated from the model proposed by Sih and Barlow [33].

(6) $$\frac{\lambda_{Powder}}{\lambda_{f}}=\left(1-\sqrt{1-\varphi }\;\right)\left(1+\frac{\varphi \lambda_{r}}{\lambda_{f}}\right)+\sqrt{1-\varphi }\;\left(\frac{2}{1-\frac{\lambda_{f}}{\lambda_{s}}}\left(\frac{1}{1-\frac{\lambda_{f}}{\lambda_{s}}}\ln\left(\frac{\lambda_{s}}{\lambda_{f}}\right)-1\right)+\frac{\lambda_{r}}{\lambda_{f}}\right)$$

where $\rho_{powder}$, $\rho_{solid}$, and φ represent powder density, solid density, and powder porosity, respectively. Likewise, $\lambda_{powder}$ represents powder conductivity, $\lambda_{s}$ is termed as solid conductivity, and $\lambda_{f}$ is the thermal conductivity of Argon gas, which ensures powder supply and protection to the melt pool during consolidation; whereas, $\lambda_{r}$ is radiative conductivity and it may be calculated with Equation (8) for desired material [7].

(7) $$\lambda_{r}=4F\sigma T^{3}x_{r}$$

where σ represents the Stefan–Boltzmann constant; $x_{r}$ is the average particle diameter; *T* is the temperature of the powder particle; and *F* is known as the view factor, which is taken as 1/3 and represents the fraction of thermal radiation emitted from a surface that is intercepted by another surface. Calculated powder density and thermal conductivity for FEM simulation are shown in Figure 6.

Moreover, radiation from the powder bed surface is emitted from individual particles as well as from cavities present in the powder bed or stream and is affected by temperature and composition. Powder emissivity $\varepsilon_{powder}$ is calculated from a previous model proposed by Sih and Barlow [33].

(8) $$\varepsilon_{powder}=A_{H}\varepsilon_{H}+{\left(1-A_{H}\right)}_{\varepsilon_{bulk}}$$

where $A_{H}$ shows a fraction of powder covered by hot particles in mm2 and $\varepsilon_{H}$ denotes the emissivity of holes, which is expressed as

(9) $$f=\frac{\mathrm{hole}\;\sec\mathrm{tion}}{\mathrm{total}\;\mathrm{hole}\;\mathrm{surface}}$$

(10) $$\varepsilon_{H}=\frac{\varepsilon_{\mathrm{bulk}}}{\varepsilon_{\mathrm{bulk}}+f\left(1-\varepsilon_{\mathrm{bulk}}\right)}=\frac{\varepsilon_{\mathrm{bulk}}\left[2+3.082{\left(\frac{1-\varphi }{\varphi }\right)}^{2}\right]}{\varepsilon_{\mathrm{bulk}}\left[1+3.082{\left(\frac{1-\varphi }{\varphi }\right)}^{2}\right]+1}$$

(11) $$A_{H}=\frac{\mathrm{holessurface}}{\mathrm{total}\;\mathrm{surface}}=\frac{0.908\varphi^{2}}{1.908\varphi^{2}-2\varphi +1}$$

The parameters in the equation can be determined experimentally or theoretically, based on the properties of the material and the process conditions. The equation can be used to optimize the design and operation of processes involving powder beds, by predicting the radiative heat transfer and the resulting temperature distribution in the deposited powder.

### 3.3. Heat Source Configuration

Appropriate heat source modeling is very crucial to replicate heat distribution and melt pool characteristics as in the actual consolidation process. Goldak’s moving heat source is suited well as it counts heat gradient at the melt pool surroundings too [34]. Gaussian heat distribution is realized to obtain adequate resemblance to the physical laser consolidation phenomenon occurring in the DED process [35].

(12) $$q\left(x,\;y,z,\;t\right)=\frac{6\eta \sqrt{3}Q}{abc\pi \sqrt{\pi }}\exp\left(-\frac{3{\left(x-vx.t\right)}^{2}}{a^{2}}-\frac{3{\left(y\right)}^{2}}{b^{2}}-\frac{3z^{2}}{c^{2}}\right)$$

where a,b,c represent heat source shape in transverse penetration and longitudinal direction, which is taken as constant to form a uniform circular spot. *Q*, v, t, and η represent the laser power, velocity, time, and laser absorptivity, respectively. Absorption efficiency of 48% is selected as it is obtained experimentally [36]. Melt pool configuration is closely associated with the implemented heat source model [7]. The simulated melt pool configuration is validated with experimental results to provide the adequacy of the numerical model.

### 3.4. Transient Materials Deposition

Modeling of material deposition is much more difficult for the laser cladding process contrary to powder bed fusion (PBF) additive manufacturing where the whole layer is activated for consolidation. Overlooking the deposition strategy in simulation may compromise the accuracy of the predictive simulation. Particularly, spatial and temporal heat distribution may deviate with the velocity of powder stream. Therefore, a progressive element birth and death technique is implemented to resemble the actual cladding process in which transient activation of elements after deposited geometry is added in hundreds of steps only in the heating step.

### 3.5. Thermal Analysis

The transient temperature distribution T(x,y,z,t) is calculated by solving the 3D heat conduction equation with a hybrid scheme combined with the Newton–Raphson method and implicit method [37]. The powder material is considered isotropic for heat transfer analysis.

(13) $$\frac{\partial }{\partial x}\left(\lambda \frac{\partial T}{\partial x}\right)+\frac{\partial }{\partial y}\left(\lambda \frac{\partial T}{\partial y}\right)+\frac{\partial }{\partial z}\left(\lambda A\frac{\partial T}{\partial z}\right)+\;q=Cp\left(\frac{\partial T}{\partial t}\right)$$

where λ, q, T, cp, ρ, and *t* are the thermal conductivity, heat supplied rate, temperature material density, specific heat, and interaction time of the heat source and material, respectively. The initial temperature (25 °C) is assigned as ambient temperature ${T{}^{\circ}}^{\prime }$ conditions, as stated here.

(14) T(x,y,z,0)=T°

(15) $$T{\left(x,y,z,t\right)}_{t=0}=T_{0}\left(x,y,z\right)$$

Moreover, a small amount of heat *Q* is dissipated through convection and radiation phenomena, and it is expressed with a mathematical expression as

(16) $$Q=-\left[h_{c}\times \left(T-T_{a}\right)-\varepsilon \sigma \left(T^{4}-T_{a}^{4}\right)\right]$$

where $h_{c}$, *A*, σ, ε, T, and $T_{a}$ are convective heat transfer coefficient, surface area, Stefan–Boltzmann constant, material emissivity, and the surface temperature of solid and ambient temperature, respectively. Convective heat transfer coefficient ($h_{c}$) entirely depends on temperature and domain size. It can be further explained through the equation below:

(17) $$h_{c}=\frac{N_{u}k_{f}}{L}$$

where $N_{u}$ is the Nusselt number, L is specimen length, and $k_{f}$ stand for the fluid thermal conductivity in the above equation for powder materials in additive manufacturing [38]. Another thermal boundary condition is observed to demonstrate no heat exchange from the bottom surface of the substrate.

(18) $${\frac{\partial T}{\partial z}|}_{z=bottom\;surface}=0-\lambda$$

**Figure 7:**
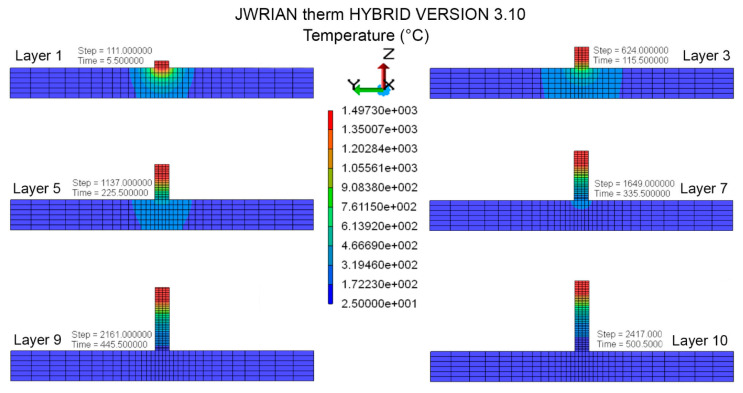
FEA transient temperature profile in middle cross-section during layer-by-layer consolidation.

Marangoni flow, which is the effect of fluid motion due to the thermo-capillary phenomenon, is completely overlooked here. The contours for temporal and spatial temperature distribution illustrate temperature differences during consolidation in the layer and substrate, respectively, as demonstrated in Figure 7. When the first layer is deposited and consolidated on the substrate, it partially melts the substrate to establish a firm connection with the substrate. A substantial increase in baseplate temperature is observed in multilayer cladding up to consolidation of the fourth layer; then, it gradually decreases in the substrate region. The highest temperature of 1497 °C is recorded during the melting of each layer, which does not surpass multilayer consolidation and shows a stable cladding process. Otherwise, nonuniform thermal gradient can significantly affect build quality. The cladding width and height used in the numerical model is consistent with experiments; fabricated layers showed a negligible difference in extreme ends, which is completely overlooked here.

Moreover, a time increment of 45 s between inter-layer deposition leaves critical repercussions on melt pool morphology, thermal gradient, and global cooling rate during the cladding process as it serves as a cooling venture simultaneously. Denlinger et al. [39] studied the effect of dwell time in the DED process for Inconel^®^625 and Ti6Al4V. An opposite trend for residual stress is observed for both materials. Nevertheless, an appropriate dwell time assists to regulate the thermal gradient based on the scan strategy [40]. Furthermore, processing conditions—e.g., scanning velocity, heat input, and solidification rate—influence the spatial temperature variation and subsequently affect grain morphology and microstructure [41]. Wolff et al. [42] evaluated laser deposition of Inconel718 and investigated the effect of cooling rate on microstructure.

### 3.6. Mechanical Analysis

A hybrid scheme is implemented contrary to the traditional implicit FEM approach; therefore, in accelerated explicit FEM, the nodal velocity and nodal displacement are calculated from the acceleration on nodes. A similar mesh scheme and element size are preferred for thermal as well as mechanical analysis for numerical compatibility. Nodal temperature data are imported for mechanical analysis, and inherent strain-based calculation is opted to perform an efficient computational solution. This stress and strain can be associated as stated by the equations below.

(19) v(t+dt)=v(t)+a(t+dt). dt

(20) du(t+dt)=v(t+dt). dt

Strain increment dε(t+dt) at the integration point are

(21) dε(t+dt)=B.du(t+dt)

where *B* is the matrix to demonstrate a relationship between nodal displacement and subsequent strain for each element. Thermal stress σ(t+dt) is calculated through the thermal elastic–plastic formulation proposed by [43].

(22) $$\sigma \left(t+dt\right)=\sigma \left(t\right)+D\left(T\right).\;\left(d\varepsilon -d\varepsilon^{T}-d\varepsilon^{p}\right)$$

where $d\varepsilon^{T}$ and $d\varepsilon^{p}$ are thermal and plastic strain, respectively; D(T) is material elastic matrix; and thermal strain increment is calculated as

(23) $$d\varepsilon^{T}=\alpha \left(T\right).dT+\frac{dD\left(T\right)}{dT}D^{-1}\left(T\right).\sigma \left(t\right).\;dT$$

Encastre boundary condition is implemented on the extreme ends of the baseplate similar to a custom-built fixture for the structural analysis, as demonstrated in Figure 2.

(24) Ux+Uy+Uz+θx+θy+θz=0

A small additional step is introduced to unclamp the substrate from the fixture; however, a spring boundary condition with a low stiffness of 1 N/mm^2^ is applied to prevent rigid body motion in unclamp situations [44].

**Figure 8:**
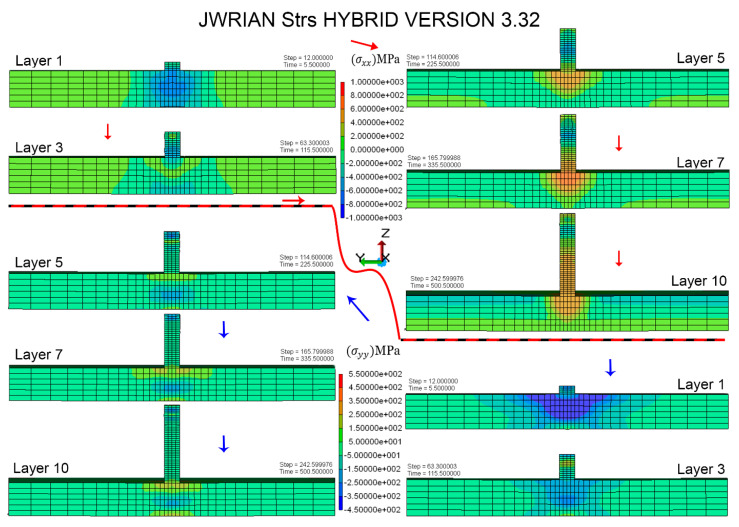
Longitudinal and transverse thermal stress distribution for subsequent layering in laser cladding simulation.

Residual stresses are produced primarily because of localized heat gradients and solidification shrinkage of the consolidated material. The magnitude and distribution of thermal stress vary over the entire part, showing a constant melting. Longitudinal and transverse stress contours for the middle cross-section of consolidated layers at respective time intervals are shown in Figure 8. The magnitude of the longitudinal component ($\sigma_{xx}$) of residual thermal stress is higher than the transverse component ($\sigma_{xx}$) mainly due to the material anisotropy and the spatial temperature distribution and coefficient of thermal expansion mismatch briefly discussed in the results and discussion section. The consolidated material and heat-affected zone (HAZ) are supposed to expand during heating, which is restricted by low-temperature surroundings. Ultimately, this caused compressive stress in the new layer and tensile stress in the underlying layers. Similarly, the material experiences a contraction in subsequent cooling steps, resulting in tensile stress in newly deposited material and compressive stress in preceding layers when the mechanical boundary condition is removed; then, the substrate can freely distort and the corresponding residual stresses are redistributed in the middle cross-section.

Since steep thermal gradients encourage residual stresses, which may compromise dimensional tolerance and geometric accuracy, a modified inherent strain method is implemented to compute residual distortion, which has significantly improved computational efficiency [45]. Mechanical analysis through the inherent strain method in FEM-hybrid can be easily implemented with substantial time savings for additive manufacturing. A short interval of 1 s is introduced to resemble the process of removing fixtures for the accurate prediction of part distortion. The numerical model is calibrated and verified by melt pool configuration, residual stress, and distortion in the substrate.

## 4. Results and Discussion

### 4.1. Melt Pool Validation

**Figure 9:**
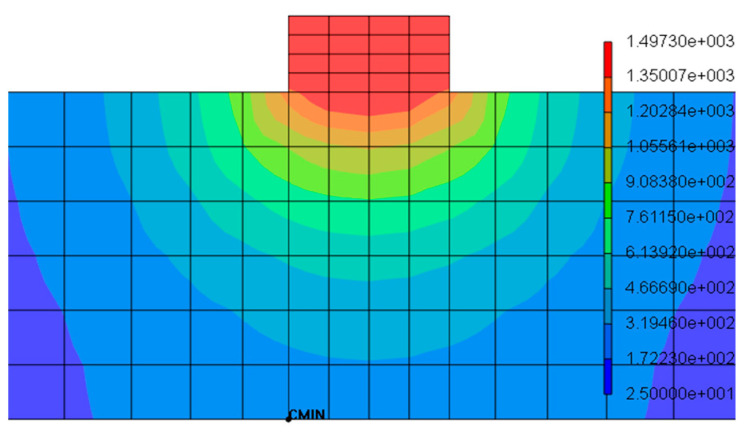
Gaussian heat distribution and melt pool configuration in complete layer consolidation.

Melt pool configuration is directly dictated by the variation energy density and plays a critical role in fabrication. The large melt pool dimensions due to high energy density introduce micro-humping, and overlapping of the adjacent deposits could result in the deterioration of flatness in consolidated layers [46]; on the other hand, low energy density leads to balling phenomena due to insufficient melting, eventually provoking poor metallurgical bonding and interlayer delamination [47]. The temperature distribution is ellipsoidal Gaussian and the temperature gradient is sufficient enough to melt the complete layer. The melt pool vicinity with clear and dense isothermal layers shows complete consolidation, as seen in Figure 9. Likewise, isothermal layers are relatively sparse and a lower thermal gradient is envisaged in the rear part of the melt pool. Melt pool width, length, and depth are quantified with the nodal temperature above the liquid’s temperature range (1350 °C), validated further with single-track multilayer cladding experiments, and relatively stable layer consolidation is obtained except on the extreme ends up to 10 layers. A negligible difference of approximately 5% is noticed in the simulation and experimental measurement of the melt pool configuration contrary to the previous findings, which claim that melt pool width is 90% of the laser beam diameter [48]. It is observed that laser-to-material stream interaction time drives melt pool shape and size during the consolidation process. Additionally, a small temperature drop is observed in the heating step, which could be attributed to the increased thermal conductivity of previously consolidated layers.

### 4.2. Temperature Distribution

**Figure 10:**
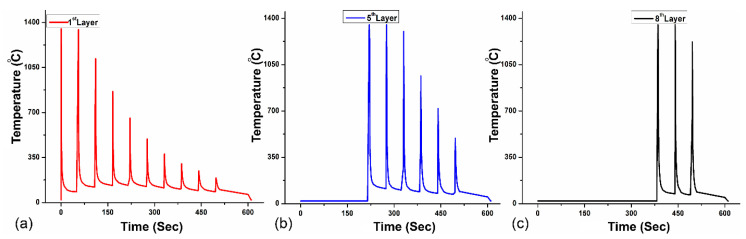
Temperature profile for subsequent layers: (**a**) first layer, (**b**) fifth layer, (**c**) eighth layer.

Accurate temperature history for each cladding layer is imperative for efficient prediction of residual stress and distortion [10]. The graphical representation (Figure 10) of time–temperature history curves for the first layer, fifth layer, and eighth layer of cladding shows convergence in thermal distribution and the subsequent peak temperatures are approximately 1497 °C. Previously consolidated layers are subjected to multiple thermal cycles during the deposition of succeeding layers. Therefore, the material on the top experiences few thermal cycles contrary to the initial layers. Thermal uniformity also contributes to the interruption in epitaxial growth of Nickel-based superalloys processed by DED [49]. Furthermore, it reduces residual strains and subsequent part deflection. Heat intensity penetrates up to the previously consolidated layer in every scan and lower layers are subjected to multiple thermal cycles during the deposition of succeeding layers on a substrate. Frequent melting provides substantial assistance to reduce porosity and side surface roughness [50]. There are no significant changes in the temperature profile for the fifth and eighth layers of deposition as temperature decreases and every layer is ensured to fuse multiple times.

Peak temperature demonstrates the distribution of heat in each layer, which exceeds the melting point of the material to show the layer is completely melted. Additive manufacturing of Inconel^®^718 is very sensitive to processing parameters; therefore, a moderate time step of 45 s for cooling is introduced to control heat gradient and reinforce the formability of Inconel^®^718 during the consolidation of the layer, and the distance between each peak shows the time interval for the next layer. Peak temperature demonstrates the distribution of heat in each layer, which exceeds the melting point of the material to show the layer is completely melted and retains the desired geometry owing to the adequate cooling step opted for Inconel^®^718 cladding.

### 4.3. Residual Stress Verification

**Figure 11:**
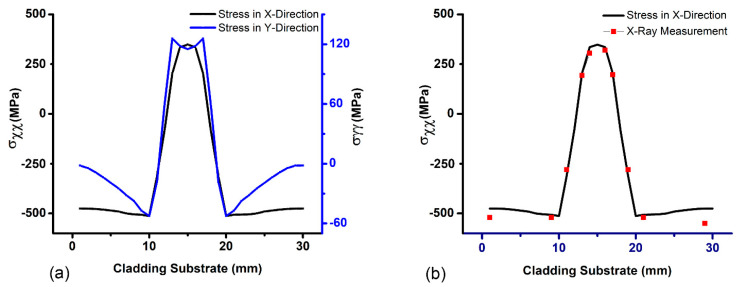
(**a**) Longitudinal and transverse residual stress. (**b**) Experimental validation.

Non-uniform heating and cooling cycles provoke residual stresses in subsequent layers and may vary in different orientations. Generally, a decrease in laser power and layer thickness can contribute to a higher Fourier number, while a lower Marangoni number leads to suppressed residual stress [51]. Moreover, the residual stress close to clad vicinity is uniform and tensile; however, compressive stresses are found on the substrate away from the deposit section in experiments as well as in simulation results [11]. Compressive residual stress is reportedly higher than tensile in the substrate, away from the consolidated geometry. The difference in tensile and compressive residual stresses is attributed to the mismatch in the coefficient of thermal expansion in substrate regions, which is principally dictated by a thermal gradient in the multilayer additive manufacturing process. It is worth noting that these compressive stresses can be beneficial for improving the overall mechanical properties of the printed part, as they can increase the strength and toughness of the substrate. However, if the compressive stresses are too high, they can also cause deformation or cracking of the substrate, which may affect the dimensional accuracy and integrity of the printed part. Residual stress variation is similar to the reported along the substrate [52]. Residual stress distribution within the layers is compressive in the center and tensile along the edges, with sizeable concentration at extreme edges as well as on the substrate interface, which also matches with the literature [53]. Experimental and FEM results are validated with more than 90% accuracy for substrate in either direction of cladding geometry. Accuracy of 93% is observed in close vicinity of deposited layers; however, 13% deviation is perceived on extreme ends, as demonstrated in Figure 11.

Longitudinal stress ($\sigma_{xx}$) is further compared with experimentally determined values of residual stress to validate FEM results in the build direction. Overall results demonstrated a precise agreement with XRD results, with a slight deviation on the extreme ends. Moreover, a considerable amount of residual stress is relaxed as a result of the distortion after unclamping the substrate. Noticeably, the longitudinal stress ($\sigma_{xx}$) component showed a significant deviation compared to numerical results obtained in a clamped position and transformed into a distortion. However, a relatively lower decrement is observed on the transverse component ($\sigma_{yy}$) upon removal of clamps. Residual stresses perpendicular to the build direction ($\sigma_{yy}$) show relatively lower values of 120 MPa compared to stresses parallel to the build direction ($\sigma_{xx}$), as shown in Figure 11a. This is because the build direction in additive manufacturing is typically the direction in which the material is deposited layer-by-layer, and this direction experiences the greatest thermal gradients and thermal cycles during the printing process. However, a relatively lower decrement is observed on the transverse component ($\sigma_{yy}$) upon removal of clamps.

### 4.4. Distortion Validation

**Figure 12:**
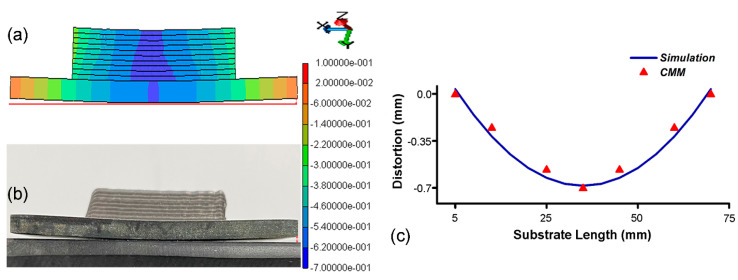
(**a**) Distortion prediction through a hybrid FEM scheme. (**b**) Experimentally determined distortion. (**c**) Comparison of FEM and CMM results.

The resulting distortion is primarily due to thermal expansion and contraction mismatch between the deposited material and the substrate. The substrate may have a different coefficient of thermal expansion than the deposited material due to variations in thermal gradient in both regions, which can cause differential expansion and contraction during the heating and cooling cycles, eventually leading to part-scale distortion. It is observed that, after clamp removal, the overall residual stresses redistributed and slightly decreased over the entire part. The substrate of the cladding geometry distorts slightly from the middle as a result of this stress redistribution. Experimental results show a deflection of 0.68 ± 0.01 mm from the middle, and an identical distortion configuration from numerical estimation is visible in Figure 12a,b. Numerical results are quite favorable with experiments with 5% variation, and it is well understood that the deformation is due to the inherent shrinkage of consolidated layers, which led to deflection as a result of residual stresses accumulated in the substrate right below the cladding.

A quantitative comparison for substrate distortion is performed as demonstrated in Figure 12. The direction of part distortion yields in the cladding direction is significant under the deposited layers. The proposed hybrid FEM approach has been carefully validated with experimental results, which show an overall 95% agreement in substrate distortion validation. Overall prediction quality proved the efficacy of the proposed hybrid FEM scheme.

## 5. Conclusions

In the proposed work, a hybrid FEM scheme comprising explicit and implicit algorithms is implemented for the analysis of thermomechanical phenomena in multilayer additive manufacturing of Inconel^®^718. Numerical results are validated with experimentally determined results effectuated with XRD and CMM. The following conclusions were drawn from the 10 layers of Inconel^®^718 cladding.

1. Processing conditions—e.g., applied energy density, scan velocity, material feed, and layer interval time—are adequate to achieve consolidated temperature (1497 °C) above the melting point and excellent formability with a layer thickness of 0.7 ± 0.1 mm throughout the fabrication.
2. Transient material deposition with exclusive powder modeling can yield precise results for FEM simulation for thermomechanical process evaluation in multilayer cladding.
3. The simulated melt pool with Gaussian heat distribution is consistent with the consolidated layers and penetration of heat to fuse powder particles adequately.
4. Residual stresses in the transverse direction of the build plate were approximately 373 ± 5 MPa in the cladding vicinity, tensile in nature, decreased gradually, and transitioned to compressive stress away from the consolidated layers. FEM results show a good agreement with experimental results collected with XRD measurement.
5. Further, substrate distortion caused by the residual stresses resulted in a 0.68 ± 0.01 mm warpage of the substrate from the middle of the part. The magnitude and pattern of the distorted substrate provided quantitative analysis and were found consistent with the simulation results.

The proposed FEM scheme with material modeling and transient material deposition is a reliable and accurate method for the estimation of residual stress and distortion in multilayer additive manufacturing. The results of this study will set forth a route to the industrial application as well as the design and fabrication of Inconel^®^718 with the DED-AM scheme. Future research could extend the proposed numerical scheme to more complex parts, thereby providing a more comprehensive analysis of additively manufactured Inconel^®^718 parts. The support structure in complex metallic fabrications is very important for quality manufacturing, and support structure optimization is one of the significant limitations in the JWRIAN-hybrid so far.

## Figures and Tables

**Table 1:** Nominal chemical composition of Inconel^®^718 Ni-based superalloy.

Element	Ni	Cr	Fe	Nb	Mo	Ti	Al	Co	Others
(wt%)	Bal.	17.5	19.4	5.0	3.17	1.07	0.68	0.2	-----

**Table 2:** Laser cladding process parameters in experiments.

Laser Power	Cladding Velocity	Defocusing Distance	Laser Emitting Mode	Frequency	Powder Feeding Rate	Carrier Gas Flow	Lift Distance
720 W	4 mm/s	+25 mm	Pulse	100 Hz	8 g/min	6 L/min	0.65 mm

**Table 3:** Temperature-dependent material properties for solid Inconel^®^718.

Temperature (°C)	25	100	300	500	700	1100
Density (Kg/m^3^)	8577.81	8555.83	8490.97	8417.53	8336.12	8097.31
Thermal Conductivity W/(m × °C)	10.2111	11.3806	14.1563	16.7501	19.2769	27.1181
Specific Heat J/(Kg × °C)	412	422	457	486	518	835
Coefficient of Thermal Expansion m/(m × °C)	1.1 × 10^−5^	1.1 × 10^−5^	1.2 × 10^−5^	1.3 × 10^−5^	1.4 × 10^−5^	1.8 × 10^−5^
Young Modulus (GPa)	208	200	194	173	171	140
Yield Strength (MPa)	700	650	600	500	560	480
Poisson Ratio	0.31155	0.3135	0.31875	0.32411	0.32958	0.35049

## Data Availability

Owing to an ongoing project, the simulation and experimental data to reproduce the findings might not be available at this stage.
